# KIR- Ligand Interactions in Hypertensive Disorders in Pregnancy

**DOI:** 10.3389/fimmu.2022.868175

**Published:** 2022-07-15

**Authors:** Katarzyna Stefańska, Martyna Tomaszewicz, Joanna Dębska-Zielkowska, Dorota Zamkowska, Karolina Piekarska, Justyna Sakowska, Maciej Studziński, Bogusław Tymoniuk, Przemysław Adamski, Joanna Jassem-Bobowicz, Piotr Wydra, Katarzyna Leszczyńska, Renata Świątkowska-Stodulska, Sebastian Kwiatkowski, Krzysztof Preis, Piotr Trzonkowski, Natalia Marek-Trzonkowska, Maciej Zieliński

**Affiliations:** ^1^ Division of Gynecology and Obstetrics, Medical University of Gdansk, Gdańsk, Poland; ^2^ Department of Medical Immunology, Faculty of Medicine, Medical University of Gdańsk, Gdańsk, Poland; ^3^ Department of Immunology and Allergy, Medical University of Lodz, Łódź, Poland; ^4^ Department of Neonatology, Faculty of Medicine, Medical University of Gdańsk, Gdańsk, Poland; ^5^ Department of Endocrinology and Internal Medicine, Faculty of Medicine, Medical University of Gdańsk, Gdańsk, Poland; ^6^ Department of Obstetrics and Gynecology, Pomeranian Medical University of Szczecin, Szczecin, Poland; ^7^ International Centre for Cancer Vaccine Science Cancer Immunology Group, University of Gdansk, Gdańsk, Poland; ^8^ Laboratory of Immunoregulation and Cellular Therapies, Department of Family Medicine, Medical University of Gdańsk, Gdańsk, Poland

**Keywords:** hypertensive disorder complicating pregnancy, KIR HLA ligand, natural killer (NK), immunology, gestational hypertension (GH), preeclampsia (PE)

## Abstract

**Hypothesis:**

The activity of natural killer (NK) cells is considered an important factor for the tolerance of the fetus during pregnancy. The complications of pregnancy, such as hypertensive disorders (HDP), may be therefore associated with this immune compartment.

**Methods:**

The current study included 41 pregnant women diagnosed with HDPs (Gestational Hypertension; GH or Preeclampsia; PE) and 21 healthy women. All the patients were under continuous obstetric care during the pregnancy and labour. The number of mother-child mismatches within killer immunoglobulin-like receptors (KIRs), their ligands [MM], and missing KIR ligands [MSLs] was assessed. KIRs and their ligands were assessed with Next Generation Sequencing (NGS) and Polymerase Chain Reaction Sequence-Specific Oligonucleotide (PCR-SSO) typing. The subsets of NK cells were assessed with multicolor flow cytometry and correlated to the number of MSLs.

**Results:**

The number of MSLs was significantly higher in HDP patients when compared to healthy non-complicated pregnancy patients. Some MSLs, such as those with 2DS2 activating KIR, were present only in HDP patients. The percentage of CD56+CD16-CD94+ NK cells and CD56+CD16-CD279+ NK cells correlated with the number of MSLs with inhibiting KIRs only in healthy patients. In HDP patients, there was a correlation between the percentage of CD56-CD16+CD69+ NK cells and the number of MSLs with inhibiting and activating KIRs. As compared to the healthy group, the percentage of CD56+CD16-CD279+ NK cells and CD56-CD16+CD279+ NK cells were lower in HDP patients. HDP patients were also characterized by a higher percentage of CD56+CD16+perforin+ NK cells than their healthy counterparts.

**Conclusions:**

Patients with HDP were characterized by a higher number of MSLs within the KIRs receptors. It seemed that the number of MSLs in the healthy group was balanced by various receptors, such as CD94 or inhibitory CD279, expressed on NK cells. Conversely, in HDP patients the number of MSLs was associated with the activation detected as the increased level of CD69+ NK cells.

## Introduction

Hypertensive disorders (HDP) are the most common pregnancy complication that contributes to increased mortality in both the mother and fetus (1, 2). The classification distinguishes, among others, chronic hypertension, gestational hypertension (GH), preeclampsia (PE), and preeclampsia superimposed on chronic hypertension (1–3).

The pathomechanism of preeclampsia is complex and involves the changes in the cardiovascular and coagulative systems. There is also genetic background which increases the risk. This systemic disorder may be accompanied by proteinuria, endothelial dysfunctions, organ failure, and regarding the fetus, intrauterine growth restrictions (IUGR). The pathogenesis is still unclear, however, several theories have been proposed to explain the basis of the process (4). Defective placentation and abnormal spiral artery remodeling seem to be the most rational explanation for the emerging symptoms of the disease (5). There is also a piece of evidence that pregnancy-related diseases, such as PE, may have an immunological basis as the decidual NK cells are involved in the trophoblast invasion and angiogenesis.

NK cells are the population that can be found both in the peripheral blood (pNK, predominantly CD56dim) and uterus (uNK, predominantly CD56bright), where they constitute 5%–10% and 70%–90% of all lymphocytes, respectively (6, 7). These are CD3- cells characterized by the expression of CD56 and CD16 on the surface, by which we are able to divide them into three major groups: CD56+16-, CD56+16+, and CD56-16+ as presented in **Figure 3.1**.

CD56+CD16- NK cells are perceived as cytokine producers, CD56+CD16+ and CD56-CD16+ are mainly viewed as a subpopulation with cytotoxic properties (8–10).

**Figure 1:**
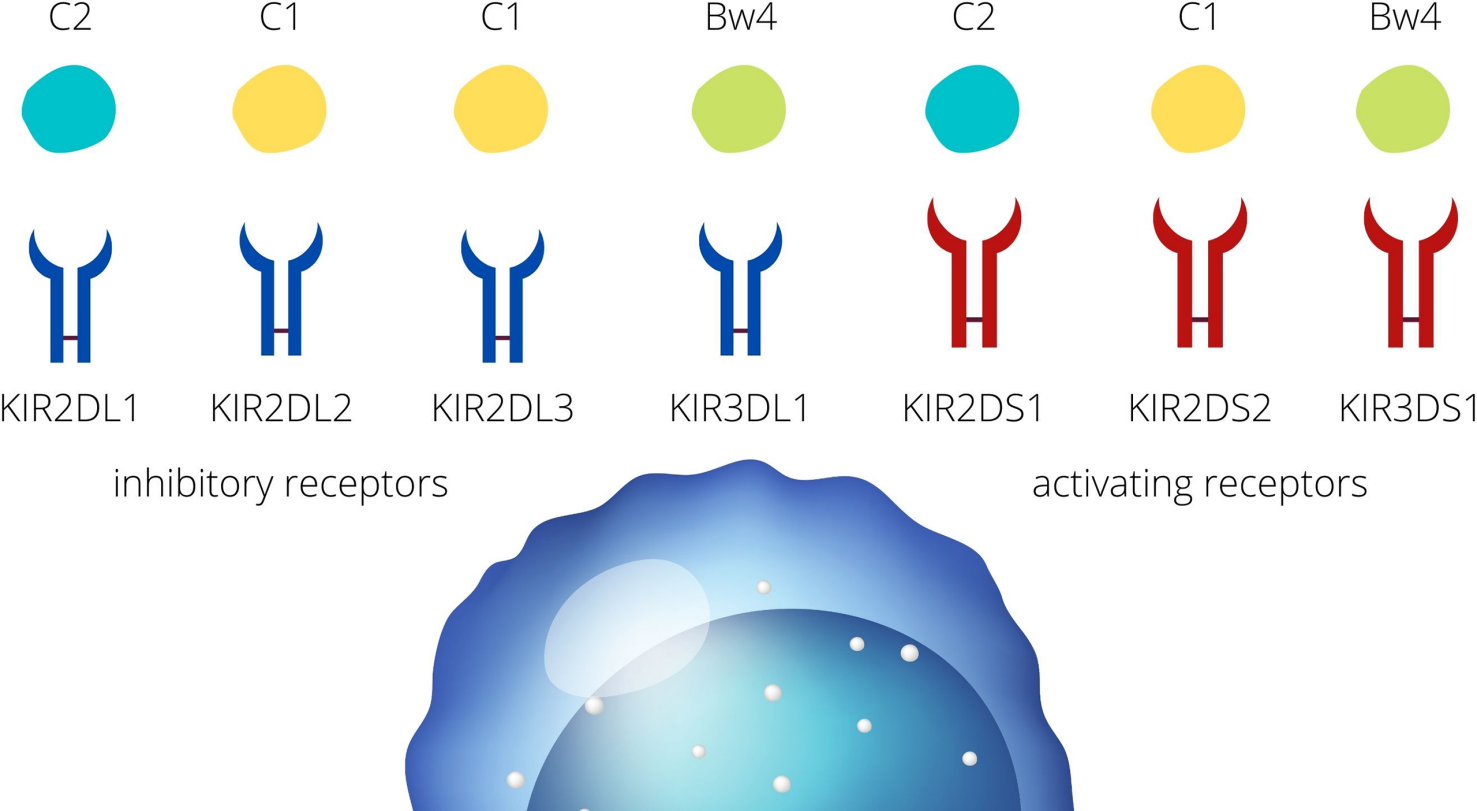
KIR- HLA interactions. KIR receptors present on the NK cells are divided into inhibitory and activating receptors. HLA ligands for the KIR receptors are classified into HLA-C1, -C2, and HLA- Bw4.

The immune system of mother adapts to changes which involves the modulation of uNK cells and are the cornerstone for the tolerance of the fetus. Killer Immunoglobulin-like receptors (KIRs) are the major players allowing NK cells to distinguish self from non-self, and therefore, license NK cells to tolerate the former and eliminate the latter. The mechanism of regulation *via* KIR receptors is based on the recognition of particular human leukocyte antigens (HLA) molecules (11). Based on the presence of particular genes, KIRs can be assigned to haplotype A or B. Haplotype A generally has no activating receptors (despite KIR2DS4, which is not active due to the very common 22 bp deletion) (12). On the contrary, the B haplotype consists mainly of activating receptors such as KIR2DS1, 2, 3, 5, and KIR3DS1 but also two inhibitory receptors, KIR2DL2 and KIR2DL5 (13). There are only some HLA class I, such as HLA-C and HLA-B, which are recognized by KIR receptors. HLA-C receptors are expressed on the trophoblast cells (extravillous trophoblast, EVT) and the ligand for KIRs may be classified into C1 and C2, according to their amino acid sequence (11, 14). On the same grounds, HLA-B receptors interacting with KIRs are classified into Bw4 and Bw6 (13, 15). KIR2DL2 (inh), KIR2DL3 (inh), and KIR2DS2 (act) bind to the HLA-C1. KIR2DL1 (inh) and KIR2DS1 (act) bind to the HLA-C2. KIR3DL1 (inh) and KIR3DS1 (act) bind the HLA-Bw4 (**Figure 1**.) (6) Subsequently, depending on the signal that is carried by the specific KIR, inhibitory or activating, NK cells become activated or remain in the resting state (6). Inhibitory receptors recognize self HLA class I and protect healthy cells from NK-mediated cell cytotoxicity (16). A discordance between the mother and child within the repertoire of KIR receptors and their ligands may be, therefore, an important reason that the mother tolerates or aborts the fetus. This process is modulated by other receptors expressed on NK cells, such as CD279 (the Programmed Death receptor 1, PD-1) or CD69, which provide inhibition or activation signals, respectively, to maintain tolerance and prevent autoimmunity (17). In this light, pregnancy-associated diseases, such as those related to HDP, may be the clinical manifestation of the erroneous regulation of NK cells *via* KIR receptors.

In this study, we aimed to indicate differences in NK cell phenotypes between the HDP and healthy patients and determine whether the KIR-ligand interactions may influence the maternal immunological tolerance of the fetus. We wanted to check if there is a change in the NK cell phenotype or interaction between KIRs and their ligands that can be treated as a potential immunological marker of hypertensive disorders occurring in pregnancy.

## Methods

### Study design

This was a single-center study in which 62 women were enrolled from all hospitalized patients between April 2018 and December 2019 at the Department of Obstetrics, Medical University of Gdansk, Poland. Patients were between 27 and 42 weeks of gestation with a singleton pregnancy with symptoms of gestational hypertension (GH), and preeclampsia (PE) but with no co-morbidities. Women with chronic secondary/essential hypertension, immunological diseases like Hashimoto’s disease, diabetes mellitus, pre-existing renal disease, intrauterine fetal death, gestational diabetes, bacteriuria, multiple pregnancies, assisted reproductive technology in pregnancy, and premature rupture of membranes, were excluded. Patients with BMI higher than 42 and those who were chronically administered aspirin or other anti-inflammatory agents were also excluded. The majority of patients were admitted beyond the 34th week of pregnancy. Those admitted prior to the 32nd week of pregnancy were admitted due to exacerbation of hypertension, worsening of the general condition, or fetal growth restriction (FGR). This study was approved by the Bioethics Committee at the Medical University of Gdansk (no. NKBBN/454/2014) and was conducted according to the principles of the Declaration of Helsinki. All participants provided written informed consent to participate in the study (10).

### Patients

**Figure 2:**
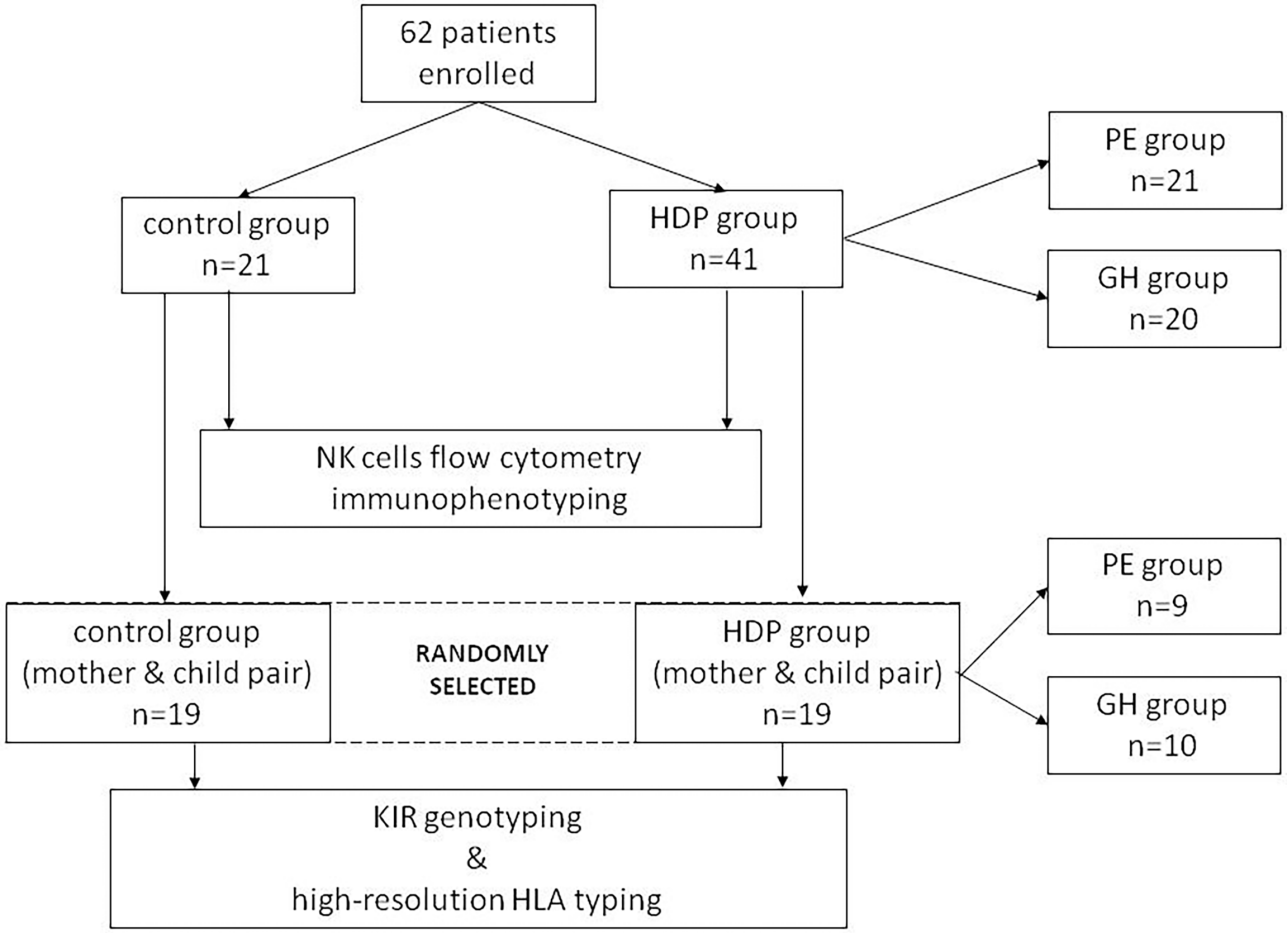
Study flow diagram.

Based on clinical and laboratory evaluations, according to the ISSHP classification (18), the patients were divided into two groups: the HDP group (n=41; PE=20; GH=21) and the control healthy group (n=21). The study flow diagram is shown in **Figure 2** and the baseline characteristics of the study population are provided in **Table 1**.

**Table 1:** Patient’s characteristics.

Patients’ status	HDP n = 41	Control n = 21	p*
**Age (years, mean ± SD)**	30 ± 4.96	31 ± 3.70	0.2
**Length of gestation (weeks) (mean± SD)**	36 **± 3.67**	39 **± 1.84**	0.0007*
**Body mass index (kg/m^2^) (median, min/max)**	30, 23/41	27, 22/36	0.0002*
**Parity**			
** 0**	30	15	NT
**1**	7	4	NT
**>1**	4	2	NT

Patients did not differ in age, but there was a statistically significant difference in two parameters: length of gestation (p=0.0007) and BMI (p=0.0002).

*Mann-Whitney U-test comparing HDP and control, p<0.05 was considered significant; NT- not tested.

HDPs were defined as systolic blood pressure ≥140 mm Hg and diastolic blood pressure ≥90 mm Hg in a previously normotensive pregnant woman after the 20th week of gestation without proteinuria or an indication of end-organ dysfunction. PE was diagnosed in patients with high blood pressure (24h blood pressure records) and new-onset proteinuria, i.e., when resting blood pressure was≥ 140/90 mmHg on two occasions that were at least 4h apart and when significant proteinuria was detected in urine samples. Proteinuria was diagnosed with a urine protein/creatinine ratio (UPCR) ≥30 mg/dl cut-off value (19). In the absence of proteinuria, PE was diagnosed based on hypertension in association with thrombocytopenia (platelet count <150,000/μL), impaired liver function (two-fold increase in blood levels of liver aminotransferases in comparison to the normal concentration), a new development of renal insufficiency (elevated serum creatinine >1.02 mg/dL), pulmonary oedema, new-onset of cerebral or visual disturbances, or uteroplacental dysfunction, including FGR. FGR was diagnosed as fetal abdominal circumference/estimated fetal weight <10th percentile combined with pulsatility index in the umbilical artery >95th percentile or pulsatility index in the uterine artery >95th percentile, or abdominal circumference/estimated fetal weight <3rd percentile, or absent end-diastolic flow in the umbilical artery (10, 11, 20).

### Sampling and preparation

Venous blood fasting samples (20ml) were collected from pregnant women at 35 ± 3 weeks of pregnancy during routine visits in the outpatient clinic between 9.00 a.m. – 10.00 a.m. Oral swabs from neonates were taken at the obstetrics ward within 2 days of the birth.

The samples of blood from mothers and the swabs from neonates were used for the isolation of DNA to study KIR receptors and HLA antigens. The remaining samples of blood from mothers were ficolled to obtain peripheral blood mononuclear cells (PBMC) for flow cytometry. The sera were centrifuged from blood samples taken without anticoagulant and stored at -80°C until use.

### Flow cytometry

**Figure 3:**
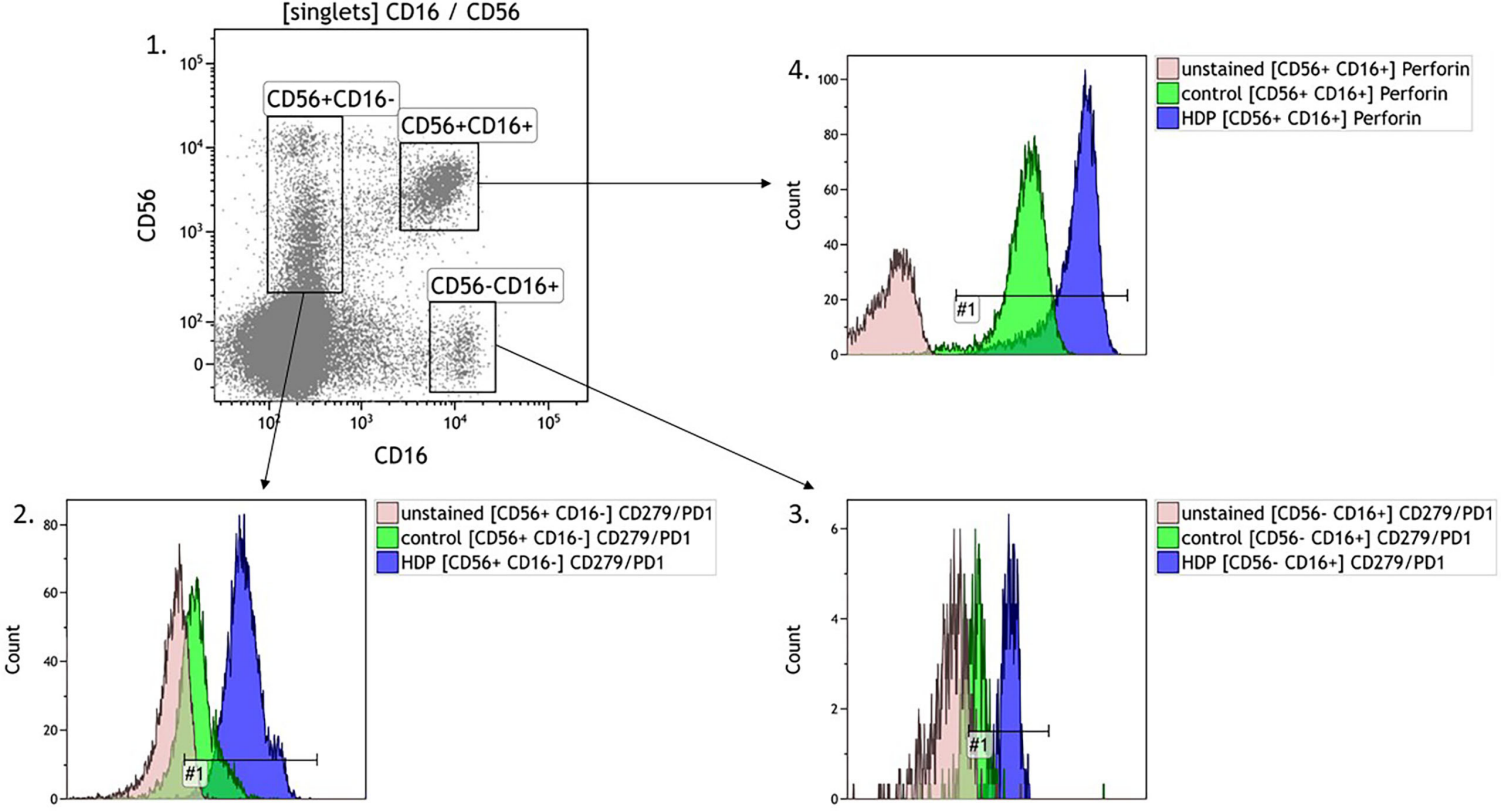
Dot plot analysis, CD16 and CD56 antigens on the NK lymphocyte population [3.1]. Expression of CD279, CD94 and perforin in NK subsets from HDP patients (blue) and healthy control patients (green). The cut-off value [#1] was established for the statistically significant parameters: [3.2] CD279 on 56 + 16-, [3.3] CD279 on CD56-16+ NK cells, and [3,4] perforin in CD56+16+NK cells, based on the unstained negative sample signal as a reference [beige].

PBMC were separated by Ficoll-Paque density gradient centrifugation. Cells were washed in phosphate-buffered saline (PBS), suspended in a small amount of PBS, and stained according to the manufacturer’s protocol using fluorochrome conjugated monoclonal antibodies. MAbs against the surface antigens were used in the following combination: CD3 (UCHT1), CD56 (NCAM16.2), CD16 (B73.1), CD279 (NAT105), CD94 (HP-3D9), CD69 (L78) (BD Bioscience, San Jose California, USA), KIR2DL4 (mAb 33), NKG2D (1D11) (BioLegend, San Diego, California, USA). Each of these antibodies was used at the recommended volume. Cells were incubated with MAbs for 30 min at room temperature, washed with PBS, and permeabilized with freshly prepared FOXP3/Transcription Factor Fixation/Permeabilization solution (60 min. at 4°C) (ThermoFisher Scientific, Waltham, Massachusetts, USA). After the permeabilization, cells were washed in perm/wash solution and stained for intracellular perforin using perforin (δG9) MAb clone (BD Bioscience) for 30 min. at 4°C. After the incubation, cells were washed and suspended in PBS for flow cytometry analysis performed with LSRFortessa Cell Analyzer. Data were analyzed with FACSDiva software (BD Bioscience). The NK cells were defined as CD56+ CD16+, CD56-16+, or CD56+CD16- (**Figure 3**). Positive cells were gated using a non-stained sample as a reference or with an FMO gating strategy. (**Supplementary Figure 3**).

### KIRs and HLA assessment

KIR- HLA assessment was performed in 38 pairs (mother and child) of which 19 were from the control group and 19 were classified into the HDP group (10 GH, 9 PE).

KIR genotyping was performed by the PCR-SSO method according to the manufacturer’s suggestions (LIFECODES KIR SSO Typing Kit, IMMUCOR). The amplicons were quantified on the Luminex 200 flow platform and analyzed using the MATCH IT for Lifecodes products software to generate KIR data. Genotyping included 16 KIR genes and two 2DS4 variants (full-length and deleted alleles): 2DS4, 3DP1, 2DP1, 3DS1, 3DL3, 3DL2, 3DL1, 2DS3, 2DS2, 2DS1, 2DL5, 2DL4, 2DL3, 2DL2, 2DL1, and 2DS5.

High-resolution typing of the HLA-A,-B,-C,-DRB1,-DQB1 were characterized using next-generation sequencing technologies (NGS) in Illumina MiniSeq (MIA FORA NGS FLEX 5 HLA Typing Kit, IMMUCOR). The obtained sequences were matched and analyzed using the MIA FORA software.

The online IPD-KIR calculator (EMBL-EBI, Hinxton, Cambridgeshire) (17) was used to classify the HLA ligands into C1/C2 and Bw4/Bw6. Two ways to define KIR-associated discordance between mother and fetus were applied: (1) missing KIR ligands (MSLs) were defined as the absence of one or more HLA epitopes in the fetus for the corresponding KIRs in the mother and (2) KIR ligand mismatch was defined as a mismatch in ligand groups between mother and fetus (KIR ligand calculator). Additionally, the KIR haplotypes were assessed (17, 21), and all mothers were assigned to the AA or Bx haplotype group.

### Statistics

Non-parametric statistics were used as suggested by data distribution. The Spearman test was performed with Statistica software (StatSoft, Polska) to assess correlations between missing activating or inhibitory KIR ligands (MSLs) and the NK cells’ phenotype parameters. The differences in the distribution of MSLs between patient groups were assessed with 2x2 tables and a χ2 test. The differences in the percentages of NK subsets between HDP and control patients were assessed with the U-Mann-Whitney and the Kruskal Wallis tests and p<0.05 was considered significant. MSLs frequencies were visualized with heatmaps with the online Heatmapper software (22).

## Results

### Major findings

The patients with hypertensive disorders (HDPs: PE and GH) presented a higher absolute number of missing KIR ligands (MSLs) than the control group. Additionally, the PE group presented a higher number of KIR-ligand mismatches than the control group. In the healthy group, the number of MSLs correlated positively with the percentage of NK cells expressing suppressive receptors.

### Missing KIR ligands

As compared to healthy controls, the number and percentage of missing KIR ligands (MSLs) were significantly higher in HDP patients (inhKIR: χ2 = 4.26 p=0.03, actKIR χ2 = 4.19 p=0.04) (**Table 2**). When HDP patients were divided into PE and GH groups, they were still characterized by a higher percentage of activating MSLs (PE: actKIR χ2 = 10.95 p=0.0009; GH: actKIR χ2 = 4.74 p=0.03). The missing ligand of KIR2DS2 (activating KIR), was present only in GH patients. The frequency of inhibitory MSLs was also significantly higher in the PE and GH groups (PE: inhKIR χ2 = 7.18 p=0.007; GH: inhKIR χ2 = 4.6 p=0.03) (**Table 2**).

**Table 2:** The distribution of missing KIR ligands that occurred in the HDP (PE and GH) and in the healthy control group.

Hypertensive Disorders of Pregnancy (HDPs)					
activating KIR			inhibitory KIR		
number of missing KIR ligands	N	%	number of missing KIR ligands	n	%
0	8	42	0	4	21
≥1	11	58	≥1	12	79
≥2	2	11	≥2	11	58
Preeclampsia (PE)					
activating KIR			inhibitory KIR		
number of missing KIR ligands	N	%	number of missing KIR ligands	n	%
0	2	22	0	1	11
≥1	7	78	≥1	8	89
≥2	1	11	≥2	6	67
Gestational Hypertension (GH)					
number of missing KIR ligands	n	%	number of missing KIR ligands	n	%
0	6	60	0	3	30
≥1	4	40	≥1	7	70
≥2	1	10	≥2	5	50
Healthy Control (HC)					
number of missing KIR ligands	n	%	number of missing KIR ligands	n	%
0	14	74	0	9	47
≥1	5	26	≥1	10	53
≥2	1	5	≥2	5	26

**Figure 4:**
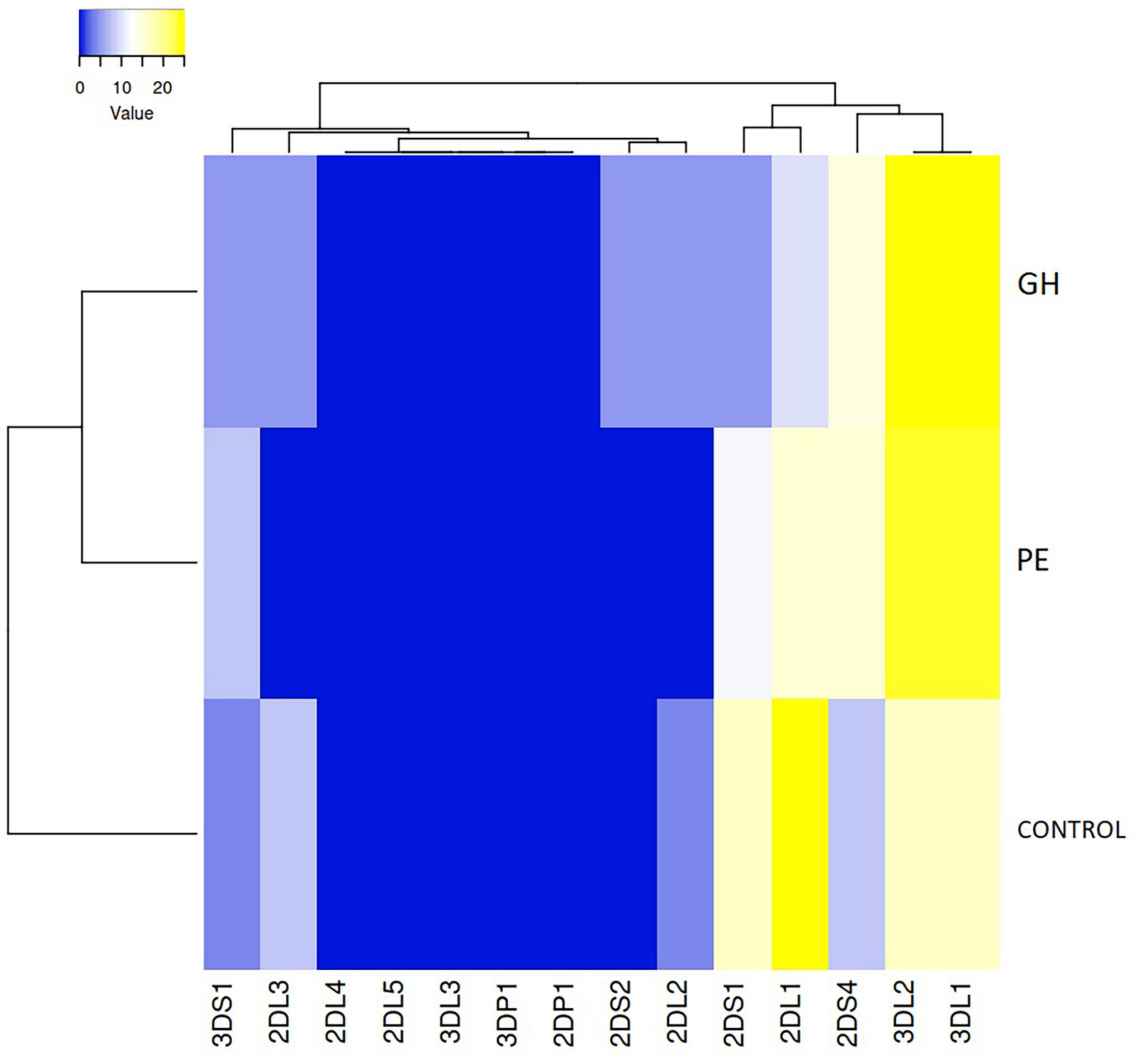
Clustering and heatmap analysis of the missing KIR ligand frequency in preeclampsia (PE), gestational hypertension (GH), and control group. Both rows and columns are clustered using the average linkage and the euclidean distance measurement method.

Clustering and heatmap analysis of the MSLs frequency revealed that 3DL1 and 3DL2 were the most frequent MSLs in HDP (in both PE and GH), 2DL1 was the most frequent MSL in the control group, while missing KIR2DS2 ligands were found only in HDP (GH) and not in the healthy control group or PE group (**Figure 4**).

### Mother-fetus KIR mismatches

**Figure 5:**
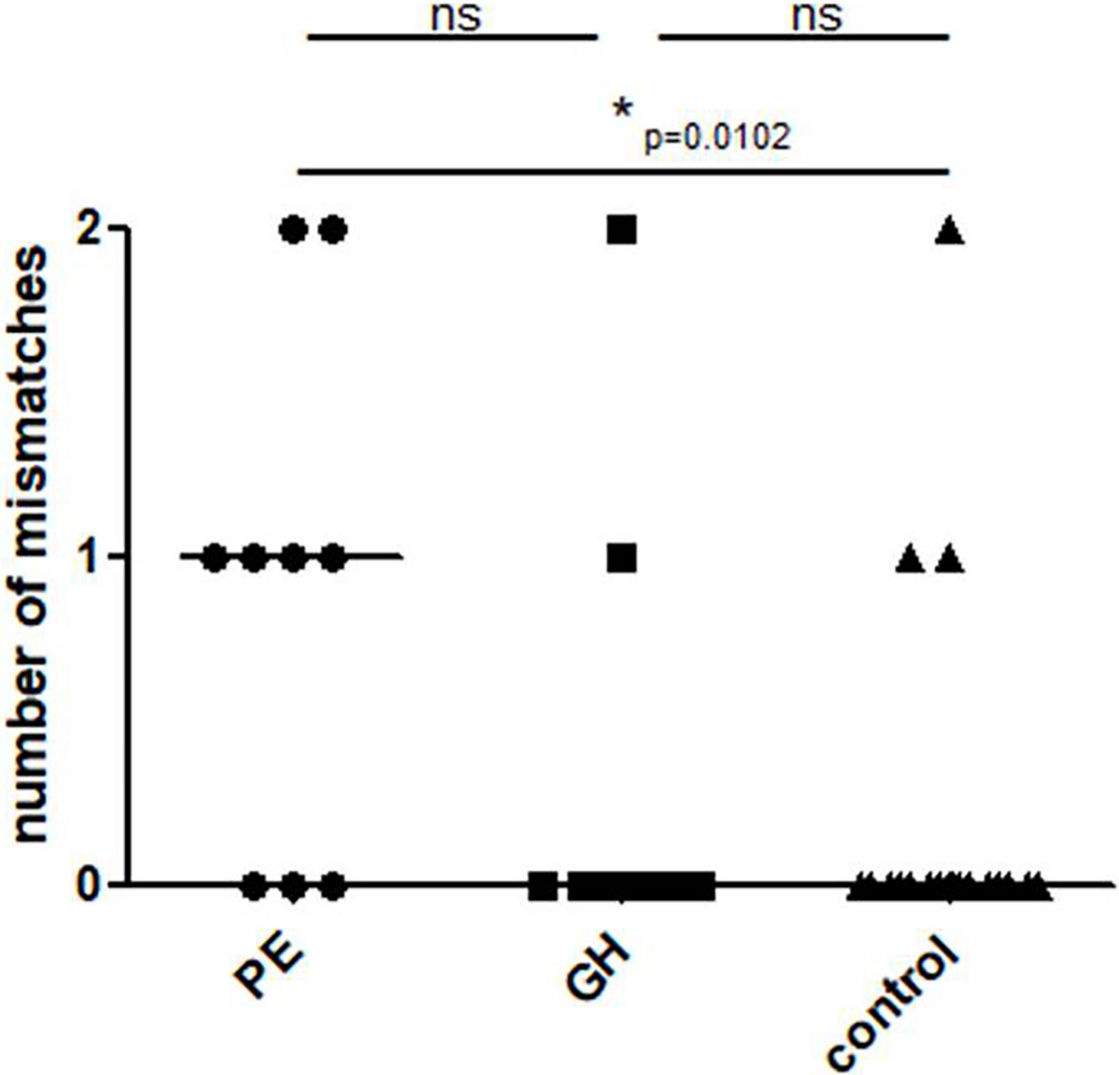
Mother-child KIR mismatches in the study groups: Control – healthy control patients, GH - Gestational Hypertension, and PE – Preeclampsia. p<0.05 is considered significant. *p<0.05; ns- not significant.

A statistically significant difference was found in the mother-fetus mismatch number between PE and the control group. (**Figure 5**, U Mann Whitney PE vs. control, p=0.0102, PE vs. GH p=0.0725; GH vs. control p=0.7801).

### NK haplotype

A comparison of the distributions of the AA to Bx KIR phenotypes in HDPs and control women revealed that there was a statistically significant difference between PE and the control group. (χ2 = 31.36, p< 0.05) (**Table 3**).

**Table 3:** The AA to Bx KIR phenotype ratio in HDPs and control women.

Hypertensive Disorders of Pregnancy (HDPs)			
PE		GH	
AA	Bx	AA	Bx
22% (n=2)	78% (n=7)	50% (n=5)	50% (n=5)
Healthy Control (HC)			
AA		Bx	
47% (n=9)		53% (n=10)	

### NK phenotype correlating with MSLs

**Figure 6:**
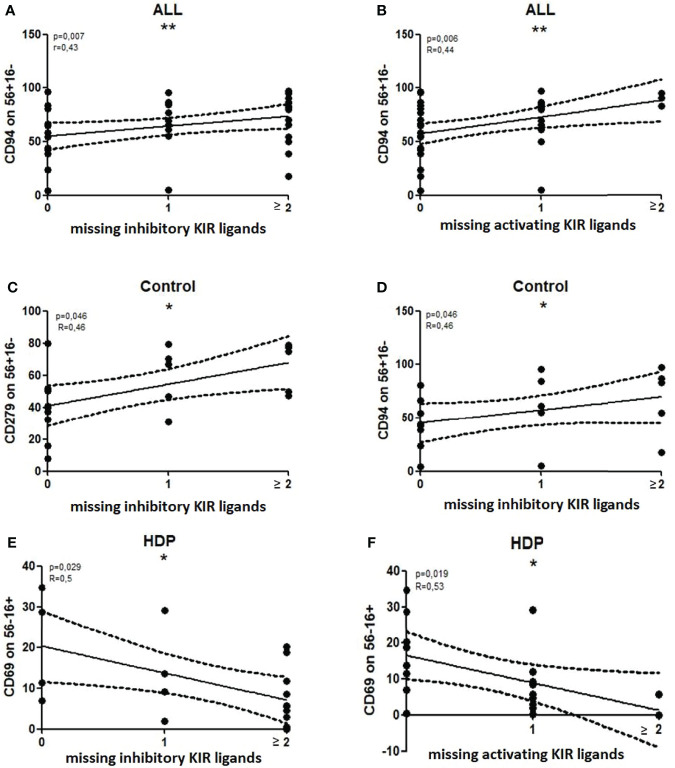
Significant correlations between the number of missing KIR ligands (MSLs) and the percentages of NK subsets. Nonparametric Spearman correlation with 95% confidence interval lines, p<0.05 is considered significant. *p<0.05; **p<0.01, ns- not significant. All – all patients, Control – healthy control patients, HDP- hypertensive disorders of pregnancy patients.

The number of MSLs within both inhibiting and activating KIRs correlated with the percentage of CD56+CD16-CD94+ NK cells when the entire cohort of patients was analyzed (Spearman’s: inhKIR: R=0.43 p=0.007, actKIR: R=0.44 p=0.006) (**Figures 6A, B**).

When the patients were analyzed according to their health status, the percentage of CD56+CD16-CD94+ NK cells correlated with the number of MSLs of inhibiting KIRs only in healthy controls (R=0.46 p=0.046) (**Figure 6D**). The other association, which was found in healthy controls only, was the percentage of CD56+16-CD279+ NK cells which also correlated with MSLs of inhibiting KIRs (R=0.46 p=0.046) (**Figure 6C**).

In HDP patients, the percentage of CD56-16+CD69+ NK cells correlated with the number of MSLs, in both inhibiting and activating KIRs (Spearman’s: inhKIR: R=0.50 p=0.03, actKIR: R=0.53 p=0.02) (**Figures 6E, F**).

### NK subsets and HDP

**Figure 7:**
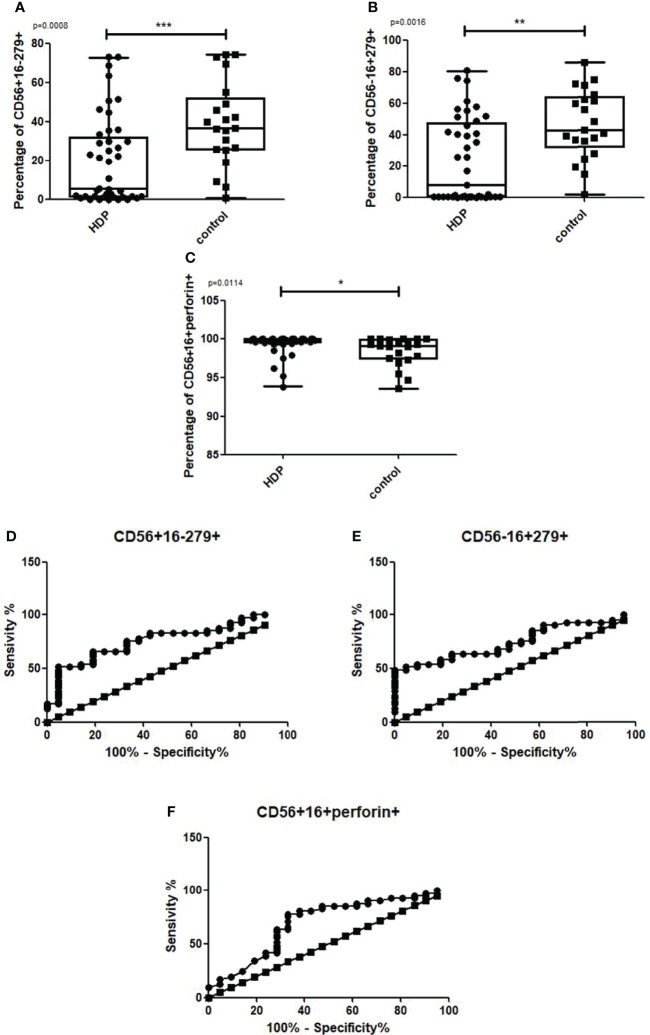
Box and whiskers graphs and ROC curves presenting statistically significant parameters that differ between the HDP and the control group. CD279 on CD56+16-, CD56-16+ and perforin in CD56+16+ NK cells. Statistics performed with the U Mann Whitney test, p<0.01,***p<0.001.

As compared to the healthy group, the percentage of CD56+CD16-CD279+ NK cells (**Figure 7A**: U-Mann-Whitney: p=0.001) and CD56-16+279+ NK cells (**Figure 7B**: U-Mann-Whitney: p=0.002) were lower in HDP patients. HDP patients were also characterized by a higher percentage of CD56+CD16+perforin+ NK cells (**Figure 7C**: U-Mann-Whitney: p=0.011). The Receiver Operating Characteristic (ROC) analysis confirmed the indicative power of the differences. The sensitivity of 65.85% and specificity of 80.95% were assessed for CD56+CD16-CD279+ NK cells (**Figure 7D**) where the cut-off value was 25.24%, and the sensitivity of 63.41%, and the specificity of 76.19% were assessed for CD56-CD16+CD279+ NK cells (**Figure 7E**) where the cut-off value was 35.64%, and the sensitivity of 63.41% and specificity of 71.43% were assessed for CD56+CD16+perforin+ NK cells (**Figure 7F**) where the cut-off value was 99.69%. The Area Under ROC Curve (AUC) was established on the level 0.745 for CD56+16-279+, 0.747 for CD56-16+279+ and 0.698 for CD56+16+perforin+.

**Figure 8:**
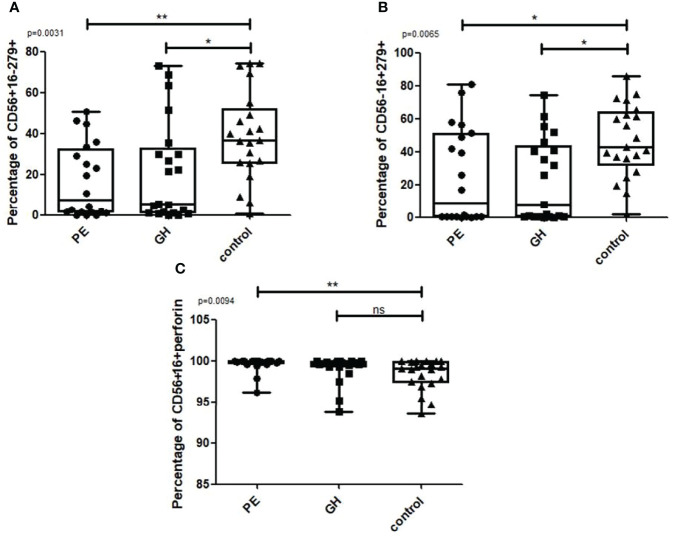
Box and whiskers graphs show differences when the HDP group is divided into PE and GH. Statistics performed with the Kruskal Wallis test, p<0.05 is considered significant. *p<0.05; **p<0.01, ns- not significant.

We divided the HDP group into preeclampsia (PE) and gestational hypertension (GH) patients. The percentage of CD56+CD16-CD279+ NK cells (**Figure 8A** Kruskal Wallis: p=0.0031; U Mann Whitney PE vs control, p=0.002; GH vs. control, p=0.0068) and CD56-CD16+CD279+ NK cells (**Figure 8B** Kruskal Wallis: p=0.0065; U Mann Whitney PE vs. control, p=0.0137, GH vs. control, p=0.0031) were significantly lower in PE and GH group than in healthy controls. PE patients were also characterized by a higher percentage of CD56+CD16+perforin+ NK cells than the control group, GH patients showed no significant difference (**Figure 8C** Kruskal Wallis: p=0.0094; U Mann Whitney PE vs. control, p=0.0052).

### 2DS4 deletion

We found no difference in the frequency of 2DS4 deletion between the HDP (PE and GH) and the control group. Additionally, there was no correlation between the 2DS4 deletion and the number of missing KIR ligands within the whole population (**Supplementary Data**).

## Discussion

In the current work, we hypothesized that the onset of hypertensive disorders in pregnancy may be associated with missing KIR ligands defined as the absence of one or more fetal HLA epitopes for the corresponding KIRs in the mother (MSLs), but also with the mismatches (incompatibilities) between KIR gene polymorphism in mothers and the HLA types in fetuses (23). Indeed, we have found that as compared to the control group, HDP patients presented a higher absolute number of missing KIR ligands. Additionally, the MSL that occurred exclusively in the HDP group (GH) was the one with activating KIR (2DS2). This might be associated with improper exaggerated activation of NK cells in HDP disorders. There was a correlation between the number of MSLs and the level of suppressive receptors on NK cells but this trend referred only to the healthy group. There was a significantly higher percentage of CD56+16- NK cells expressing CD94 and suppressive CD279 receptors in healthy controls when compared to HDP patients. The number of KIR-ligand mismatches was significantly higher in the PE group than in the control group.

The research conducted previously in solid organ transplantation patients suggested that KIR genotyping should be performed in every patient on the waiting list for the transplantation. Also, it seems that the presence of KIR- HLA type I mismatches may promote the CD4+ and CD8+ T cells activation and lead to graft damage and, as a consequence, its rejection (13). Conversely, KIR-HLA mismatches may have a positive influence in the situation of hematopoietic stem cells transplantation (HSCT) from mismatched donors. In patients with leukemia, alloreactive mismatched KIR display anti-leukemia activity in graft vs. leukemia mechanism (GvL) (24). There are reports that in preeclampsia, the activation of NK cells, probably induced by missing KIR ligands or the presence of activating KIRs, may promote the formation of inflammation disrupting the processes of implantation (25, 26). However, results obtained in the studies conducted on the KIR- HLA interactions are still particularly conflicting (27–30). Nevertheless, a growing body of evidence indicates that the relationship between KIR and HLA genes may be related to pregnancy complications. Understanding how KIR/HLA affects NK cell activity can help predicting clinical outcomes in pregnant women and personalize treatment. Perhaps in the future, KIR/HLA analysis in pregnant women will become a standard, as is currently in hematopoietic cell transplantation, where HLA typing and KIR genotyping are routinely used to improve patient outcomes.

Our results seem to prove that the basis of the physiological pregnancy is a balanced activation of the NK cells regulated *via* KIR receptors. The more missing KIR ligands were assessed in the healthy control patients, the more inhibitory receptors were expressed on NK cells which blocked excessive cytotoxicity. No such mechanism in HDP could have resulted in the disease onset. During physiological gestation, activated uNK cells, through the balanced secretion of inflammatory cytokines, such as interferon-gamma (IFN-γ) and tumor necrosis factor-alpha (TNF-α), create a pro-invasive environment allowing the trophoblast implantation. This is highly limited local and regulated process (20). Conversely, immune mechanisms triggering pathological changes in HDP are not well regulated and the exaggerated levels of the cytokines mediate overactivation of NK cells. Such NK cells are, therefore, active enough to stimulate the lysis of trophoblast cells inducing caspase-dependent apoptosis or *via* the release of cytotoxic granules (4, 18, 19). Damaged trophoblast cells are unable to invade spiral arteries causing insufficient placental perfusion (4, 7, 11). This imbalance at the level of NK activation can be detected and utilized as an immune marker predicting the risk of HDP. For example, we have recently proposed a cytokine-based algorithm to diagnose PE and a biochemical approach to study proteinuria in HDP mothers based on subtle systemic changes in the cytokine levels (31, 32). The current study may be a step ahead in the HDP diagnostics as the activation status of NK cells and the genetic reasons for the overactivation of these cells definitely precede the secretion of the cytokines or the changes in biochemical markers.

It seems that matching between KIRs and HLA is an important player in the onset of HDP but the final tuning can be also dependent on other receptors expressed by NK cells in mothers. NK cells of the HDP patients were more cytotoxic, as indicated by the lower expression of the inhibitory markers and higher expression of perforin. The HDP group showed a negative correlation between the number of MSLs and the CD69 receptor. The CD69 receptor is an early activation marker that plays a role in the production of proinflammatory cytokines, degranulation, and cytotoxic abilities (33, 34). Additionally, the HDP group was characterized by a higher number of perforin positive 56 + 16+ NK cells which are engaged in the perforin-mediated cell death (19).

Overactivity of NK cells expressing the CD69 receptor may be compensated by the expression of the CD94 receptor. This is implied by our results, as the percentage of NK cells expressing CD94 increased with the number of MSLs in HDP mothers, however, the differences were insignificant (35) (**Supplementary Figures 1A, B**). Our research did not include the determination of the NKG2 receptor, which binds CD94 forming homo- or heterodimers. Such interactions determine whether inhibitory or activating signals would be transmitted. The suppressive signals mediated by the CD94 receptor, which regulate proper activity of NK cells and allow healthy pregnancy, are not the only single mechanism of regulation. A very important path of the regulation of NK activity and, therefore, the severity of HDP may be associated with immune checkpoint inhibitors, as the interaction of one of the best-known immune checkpoints, PD-1 (CD279) with PD-ligands, inhibited NK cytotoxicity (21). Indeed, increasing percentage of NK cells expressing CD279 was associated with a lower risk of HDP in our work.

Summing up, when compared to healthy controls, HDP patients showed a higher number of mismatches and MSLs. These results indicate that the diagnostics based on KIR receptors may define risk markers for hypertensive disorders in pregnant women. The coexistence of the fetus and mother during pregnancy may be linked to the unresponsiveness of the mother’s immune system to the fetal antigens controlled by NK cells with particular suppressive immunophenotype. The match between fetal HLA and the mother’s KIR receptors seems to play an important role in the regulation of NK cells. Nevertheless, other signals such as those mediated *via* checkpoint inhibitor PD-1, should also be considered as fine-tune regulators of NK activity in pregnancy and HDP.

## Data Availability Statement

The raw data supporting the conclusions of this article will be made available by the authors, without undue reservation.

## Ethics Statement

The studies involving human participants were reviewed and approved by Independent Bioethics Committee for Scientific Research at Medical University of Gdańsk. Written informed consent to participate in this study was provided by the participants’ legal guardian/next of kin.

## Author Contributions

Conception and design of the study: MJ, KS, JD-Z, KP, MZ, and PT. Database: MJ, KS, JD-Z, KP, JS, MS, BT, and MZ. Validation: PA, JJ-B, JS, PW, KL, RŚ-S, and SK. Statistical analysis: MJ, KS, JD-Z, KP, and JS. Visualization: MJ, JD-Z, MS, and PW. Methodology: MJ, KS, JD-Z, KP, MS, BT, JJ-B, and MZ. Writing—original draft preparation: KS, MJ, JD-Z, KP, JS, MS, BT, PA, JJ-B, PW, KL, RŚ-S, SK, KP, PT, NM-T, and MZ. Writing—review, and editing: KS, MJ, JD-Z, KP, JS, MS, BT, PA, JJ-B, PW, KL, RŚ-S, SK, KP, PT, NM-T, and MZ. Supervision: KS, KP, PT, NM-T, and MZ. Funding acquisition: KS, KP, NM-T, and PT. KPi: conception and design of a study, database, statistical analysis, methodology, writing, original draft preparation, writing- review and editing, KPr: writing, original draft preparation, writing- review and editing, supervision, funding acquisition. All authors contributed to the article and approved the submitted version.

## Funding

This study was supported by the Polish National Science Center (2014/15/B/NZ5/03499) and the Medical University of Gdańsk (664/272/61/71-1403).

## Conflict of Interest

The authors declare that the research was conducted in the absence of any commercial or financial relationships that could be construed as a potential conflict of interest.

## Publisher’s Note

All claims expressed in this article are solely those of the authors and do not necessarily represent those of their affiliated organizations, or those of the publisher, the editors and the reviewers. Any product that may be evaluated in this article, or claim that may be made by its manufacturer, is not guaranteed or endorsed by the publisher.

## Abbreviations

CD56bright, a high expression of CD56 receptor; CD56dim, a low expression of CD56 receptor; EVT, extravillous trophoblast; GH-, Gestational Hypertension; HDP, Hypertensive Disorders of Pregnancy; HLA, Human Leukocyte Antigen; ITIM, immunoreceptor tyrosine-based inhibitory motif; IUGR, intrauterine growth restrictions; KIR, Killer Immunoglobulin-like Receptor; MM, Mismatch; MSL, Missing KIR Ligand; NK, Natural Killer T lymphocytes; PE, Preeclampsia.
