# Supplementary material for: KIR- Ligand Interactions in Hypertensive Disorders in Pregnancy
Source: Front Immunol. 2022 Jul 15;13:868175. doi: 10.3389/fimmu.2022.868175 (PMC9336683; doi:10.3389/fimmu.2022.868175)
Supplement: Supplementary file 1 [file DataSheet_1.pdf]

Supplementary Data

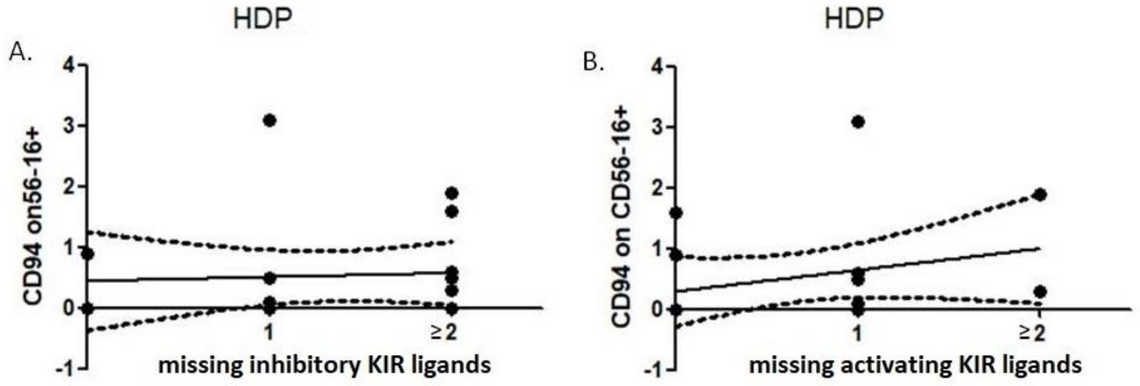

Figure 1. CD94 on CD56-16+ NK cells and correlation with missing inhibitory and activating KIR ligands. Nonparametric Spearman correlation with 95% confidence interval lines,

2DS4 deletion

There are no significant differences within the deletion/full length 2DS4 gene occurrence between the HDP and control group (U Mann- Whitney test, p=0.74), nor in the comparison of the PE, GH, and control group (Kruskal Wallis test, p=0.77). Moreover, no correlation was found between the number of missing KIR ligands and deletion/full length 2DS4 gene occurrence in the whole population (U Mann Whitney test, p=0.74).

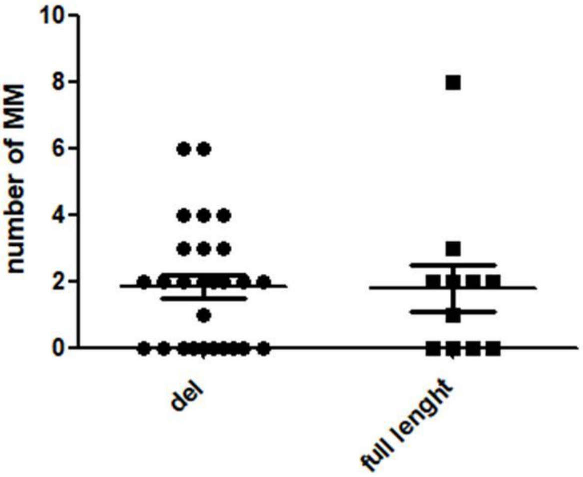

Figure 2. The correlation between the number of missing KIR ligands and deletion/full length occurence in 2DS4 gene in the whole population, U Mann Whitney test, p=0.74.
P<0.05 is considered significant.

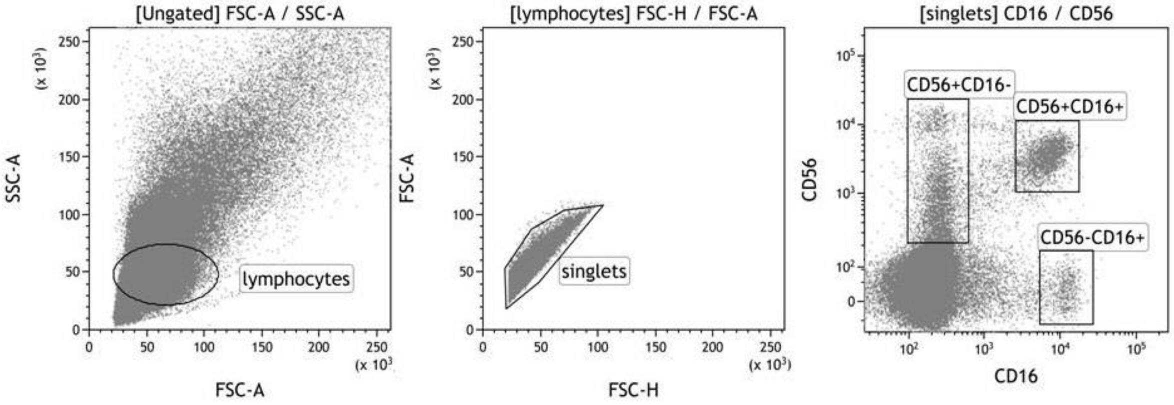

Figure 3. NK cells gating strategy.
